# *Coryanthes macrantha* (Orchidaceae: Stanhopeinae) and their floral and extrafloral secretory structures: an anatomical and phytochemical approach

**DOI:** 10.1093/aobpla/plac039

**Published:** 2022-09-02

**Authors:** Jorgeane Valéria Casique, Marcos Vinícius Batista Soares, Edilson Freitas da Silva, Tatiani Yuriko Kikuchi, Eloisa Helena de Aguiar Andrade, Alexandra Antunes Mastroberti

**Affiliations:** Programa de Pós-graduação em Botânica, Departamento de Botânica, Universidade Federal do Rio Grande do Sul, Porto Alegre, RS 91501-970, Brazil; Programa de Pós-graduação em Botânica, Departamento de Botânica, Universidade Federal do Rio Grande do Sul, Porto Alegre, RS 91501-970, Brazil; Programa de Pós-Graduação em Biodiversidade (Bionorte), Departamento de Botânica, Museu Paraense Emílio Goeldi, Belém, PA 66077-830, Brazil; Instituto de Botânica, Universidade Federal do Rio de Janeiro, Rio de Janeiro, RJ 21941-901, Brazil; Programa de Pós-Graduação em Biodiversidade (Bionorte), Departamento de Botânica, Museu Paraense Emílio Goeldi, Belém, PA 66077-830, Brazil; Programa de Pós-graduação em Botânica, Departamento de Botânica, Universidade Federal do Rio Grande do Sul, Porto Alegre, RS 91501-970, Brazil

**Keywords:** Amazon, floral fragrance, nectaries, orchids, secretory structures

## Abstract

*Coryanthes* is one of the most fascinating genera of Stanhopeinae (Orchidaceae) because of its complex pollination mechanism and the peculiar structures of its flowers. Although *Coryanthes macrantha* is widely studied, investigation of the secretory structures and floral biology is important to understand the mechanisms and ecology of pollination, which deserve attention despite the difficulties of collecting fertile material in nature. We conducted a morpho-anatomical analysis of the floral and extrafloral secretory structures of *C. macrantha* to better understand the secretory structures, contribute to the knowledge of its floral biology and/or pollination processes and understand the ecological function of these structures. The analysis revealed that *C. macrantha* has epidermal osmophores with unicellular papillae that were foraged by male *Eulaema* bees, floral nectaries in the sepals and extrafloral nectaries in the bracts. In both the floral and extrafloral nectaries, the nectar is exuded by the stomata. *Azteca* ants foraged the bract and sepal nectaries in pre-anthesis and post-anthesis. We also described the secretory epidermis of pleuridia, and the mode of secretion of osmophores and nectaries and found that they attract specific foraging agents.

## Teaser

This study brings unprecedented analyses of *Coryanthes macrantha*, a fascinating species of Orchidaceae, with wide occurrence in the Brazilian Amazon. This species present an ingenious pollination mechanism, due to the peculiar structure of the labellum. Osmophores, structures that secrete volatile substances, are located in the labellum and are responsible for attracting male Euglossini bees. The attracted bees fall into a structure like a bucket (epichile) full of a liquid, and later exit through a small lateral passage, thus removing the pollinarium. In *C. macrantha*, ants, nectaries, trichomes, osmophores, bees and stomata were observed and relationships between them were investigated.

## Introduction

The flowers of *Coryanthes*, due to their complex pollination mechanism, are among the Orchidaceae most interesting (Dodson *et al*. 1969; Dressler 1993; Gerlach 2003). This complexity is due to the morphology of the labellum of these genus, and as in the other genera in the subtribe Stanhopeinae, the labellum of the flower is subdivided into the hypochile, mesochile and epichile (Gerlach 2009). The male euglossine bees of appropriate size are attracted by specific odours and they fall into the epichile, which is filled with a liquid secreted by the pleuridia (Nazarov and Gerlach 1997; Gerlach 2011).

Species of *Coryanthes* are epiphytic herbs, with generally hanging inflorescences with fleshy, heavy, brightly coloured flowers. A striking feature is the ventrally flattened column with two prominent glands (pleuridia), which are subsquadrate to falcate, and secrete an aqueous substance (Schnepf *et al.* 1983; Gerlach 2009).

The osmophores commonly described for species of Stanhopeinae are generally located in the hypochile region and emit volatile substances as a reward for their only pollinators, male bees of the tribe Euglossini (Vogel 1990; Whitten *et al.* 2000; Gerlach 2003). The bees collect these substances by scraping the osmophores with the tarsi of the forelegs and storing them in their hind tibias; these compounds do not serve for nutrition or protection, and are presumably used as precursors in the synthesis of a sex pheromone (Vogel 1962; Van der Cingel 2001; Gerlach 2003; Bembé 2004). The use of these compounds in reproductive activities has not yet been properly confirmed (Dodson *et al*. 1969; Dressler 1982; Pansarin and Amaral 2009).

The neotropical genus *Coryanthes* includes about 60 species, 22 of which are cited for Brazil and 18 for the Brazilian Amazon (Flora e Funga do Brasil 2020). Despite the widespread occurrence of this genus in the Brazilian Amazon, studies remain few, due to the difficulty in collecting fertile specimens in nature as well as cultivating them, and to their intrinsic mutualistic relationship with ants (Gerlach 2011; Engels *et al.* 2017). In *Coryanthes* species, the mutualistic partnerships between ants and their plants are known.

*Coryanthes macrantha* grows in so-called ant gardens. As with other species of *Coryanthes*, symbiotic interactions with ants of the genera *Azteca*, *Camponotus* and *Crematogaster* are common (Schnepf *et al*. 1983; Gerlach 2009, 2011).

The symbiotic interaction is established from the moment that the ants are attracted by extrafloral and floral nectaries. The ants build their nests in the matrix formed by the *Coryanthes* root system, providing nutrients and fiercely protecting the plant, mainly young vegetative and reproductive organs such as flower buds, against attacks by herbivores (Gerlach 2009, 2011). Cultivation of these plants in orchidaries is difficult or unsuccessful, since interaction with these insects is essential for development and flowering of members of the genus (Dressler 1982; Gerlach 2009, 2011).

Most of the anatomical studies involving *Coryanthes* species were carried out on vegetative organs, such as the leaf anatomy of *C. macrantha* (Stern and Whitten 1999) and roots of *Coryanthes speciosa* and *Coryanthes maculata* (Pridgeon *et al.* 1983; Porembski and Barthlott 1988). In the only study of reproductive organs, Schnepf *et al*. (1983) focused on glands and the chemical composition of the exudate from pleuridia of *C. speciosa*, and characterized this structure as a hydathode but did not mention if it is active or passive.

Casique *et al*. (2018) studied the floral and extrafloral secretory structures in *Stanhopea grandiflora*, analysed the osmophores in the labellum (hypochile) and described novelties for Stanhopeinae, such as the nectaries of the bracts and sepals and the colleters in the ovary region. The authors also observed the activity of ants of the genera *Dolichoderus* sp. and *Azteca* sp. in the region of the bracts and sepals of this species.

The present study investigated anatomical, histochemical and phytochemical aspects of the floral and extrafloral secretory structures of *C. macrantha*, in order to characterize their structures, understand the dynamics and composition of the secretion. In addition, we sought to understand the ecological role of these structures in this species, mainly to provide data on their relationships with foragers.

## Materials and Methods

### Plant material and field observation

Three flowers of a single specimen of *C. macrantha* were collected from January to April, coming from Barcarena, a locality in the State of Pará (Brazil). After anthesis (around 7 am), the flowers of *C. macrantha* were duly identified and fertile samples were processed using standard techniques and deposited in the MG herbarium at Museu Paraense Emílio Goeldi, under the respective voucher MG151349.

Three specimens were subsequently cultivated in a private orchidarium under abiotic conditions similar to the natural environment of the species and, when possible, with part of the ant nest, to observe new flowering events and potential floral visitors. The flowers in pre-anthesis and anthesis were isolated by a screen (non-woven fabric), for about 1 h, we detected the production of nectar and glucose test strips were used (Glicofita Plus®, Accu-Chek Active, F. Hoffmann-La Roche Ltd.) to check the occurrence of glucose in the glandular secretions.

The species were identified by Mr. João Batista Fernandes da Silva, an expert on Amazon orchids. Foragers observed were collected and conserved in commercial ethanol 70 %, and were identified by Dr. Fernando Carvalho, an entomologist from the Museu Paraense Emílio Goeldi.

### Light microscopy

For the anatomical analyses, the material collected from the bracts (pre-anthesis), sepals (post-anthesis), labellum and column (pleuridia) were fixed in 1 % glutaraldehyde, 4 % formaldehyde and sodium phosphate buffer pH 7.2–0, 1 M (McDowell and Trump 1976), without vacuum, then dehydrated in an ascending ethanol series (10 %, 30 %, 50 %, 70 %, 90 % and 100 %) and embedded in hydroxyethyl methacrylate (Leica® historesin; Gerrits and Smid 1983). Serial cross-section and longitudinal sections approximately 3 μm thick were made using a rotary microtome (Leica Autocut) and stained in toluidine blue O (C.I. 52040) in 0.1 M sodium acetate buffer pH 4.7 (O’Brien *et al.* 1965). The permanent slides were mounted in Canadian balm and observed by light microscopy in brightfield (Leica DMR).

### Histochemical tests

Fresh material of labellum (hypochile), sepals and bracts was used for the following histochemical tests: Lugol’s reagent for starch detection (Johansen 1940); Fehling’s reagent for reducing sugars (product released by the action of enzymes such as cellulase, for example) (Sass 1951); Sudan III for lipids (Johansen 1940); neutral red under fluorescence for lipids (Kirk-Junior 1970); Nile blue sulfate for neutral and acid lipids (Cain 1947); copper acetate and rubeanic acid for fatty acids (Ganter and Jollès 1969, 1970); NADI for essential oils and resins (David and Carde 1964) and Xylidine Ponceau for total proteins (Vidal 1970). Untreated samples were also analysed, and negative control tests were carried out. Negative controls were performed in the test for lipids (Sudan III, Sudan IV, NADI reagent, Nile blue sulfate, copper acetate and rubeanic acid), for which sections were washed with a solution of methanol/chloroform/H_2_O/HCl (66:33:4:1) for 1 h at room temperature before the test was performed. Analyses were conducted and photomicrographs were taken under light microscopy in brightfield (Leica DMR), except for neutral red, which was observed under epifluorescence in UV light (excitation filter 450–490 nm). Images were acquired with a digital camera (AxioCam HRc, Zeiss) coupled to the microscope. The program ZEN Light 2012 was used for image capture. Untreated sections and negative controls of the bract and labellum can be seen in Figures S1 and S2 in the Supporting Information.

Structural nomenclature is based on Metcalfe and Chalk (1950); Withner *et al.* (1974); Fahn (1979); Vogel (1990); and Curry *et al*. (1991).

### Chemical analyses

The labellums of three fresh flowers after anthesis (around 7 am) from three specimens each were used, and the material was subjected to extraction (3 h) in a simultaneous microsystem of distillation extraction to obtain the volatile concentrates. A repetition of the analysis was performed for each specimen, using a Likens & Nickerson-type apparatus (Likens and Nickerson 1964) and pentane as the solvent (4 mL). Volatile concentrates were analysed by GC-MS, using a QP-2010-Pluschromatograph-mass spectrometer (Shimadzu Corporation, Tokyo, Japan) and a silica capillary column (Rtx-5MS, 30 m × 0.25 mm × 0.25 µm film thickness), with the aid of MS Solution software and standard libraries (Jennings and Shibamoto 1980; Adams 2007; Mondello 2011). The analysis conditions were: injector temperature 250 °C; oven temperature programming 60–250 °C (3 °C min^−1^); helium carrier gas (32 cm s^−1^) measured at 100 °C; splitless-type injection of 1.0 μL of the sample; ionization by electron impact, 70 EV; and temperatures of the ion source and transfer line 220 °C and 250 °C, respectively. Mass spectra were obtained by automatic scanning (0.3 s each), with mass fragments varying from 40 to 450 m/z. The retention index was calculated for all volatile components, using a homologous series of alkanes (C8–C20, Sigma-Aldrich) according to Van Den Dool and Kratz (1963). Constituents were identified by comparing their retention indices and mass-spectra libraries (molecular mass and fragmentation pattern), as well as consulting the mass-spectra literature. The analysis was undertaken at the Adolpho Ducke Laboratory of the Museu Paraense Emílio Goeldi.

### Scanning electron microscopy

For scanning electron microscopy (SEM), the fixed material (bract, sepals, pleuridia and labellum) was dehydrated in a graded acetone series and critical-point dried (Gersterberger and Leins 1978) with a CPD 030 Balzers dryer. The material was attached to aluminium stubs with double-sided carbon tape, metalized with gold in a BAL-TEC SCD 050 (Balzers) sputter coater and analysed using a scanning electron microscope (JEOL 6060, Japan) at the Microanalyses and Microscopy Center (CMM) of the Universidade Federal do Rio Grande do Sul (UFRGS).

### Transmission electron microscopy

The bracts were fixed in 2.5 % glutaraldehyde, 2.0 % formaldehyde in 0.1 M sodium phosphate buffer, pH 7.2, and postfixed in 1 % osmium tetroxide in 0.1 M sodium phosphate buffer, pH 7.2. The samples were then washed in the same buffer (two exchanges of 30 min per stage) and distilled water (two exchanges of 30 min per stage). The material was dehydrated in an acetone series (10 %, 30 %, 50 %, 70 %, 90 % and 100 %) for 30 min in each stage and finally for 15 min in acetonitrile. The material was first embedded in a pure acetonitrile solution (0.5 mL) and dipped every 10 min in a low-viscosity epoxy resin (Spurr 1969) until a 1:1 ratio was achieved, for 12 h. The material was transferred to a solution of resin/acetonitrile at a 3:1 ratio and was subsequently embedded in pure resin, remaining for 12 h in each stage. Embedding and polymerization were carried out in jelly capsules, with the material placed in an incubator at 70 °C for 18 h.

These blocks were sectioned approximately 70 nm thick, using an ultramicrotome (Leica Ultracut UCT) and a diamond blade. These ultrathin sections were placed on copper grids of mesh size 200 and stained using 2 % uranyl acetate in aqueous solution and lead citrate (modified from Hanaichi *et al*. 1986).

Transmission electron microscope (TEM) images were obtained at the Microanalyses and Microscopy Center (CMM) of the Universidade Federal do Rio Grande do Sul (JEOL JEM 1200 EX II).

## Results

### Morphological and ecological aspects

#### The genus Coryanthes.

**Figure 1.**
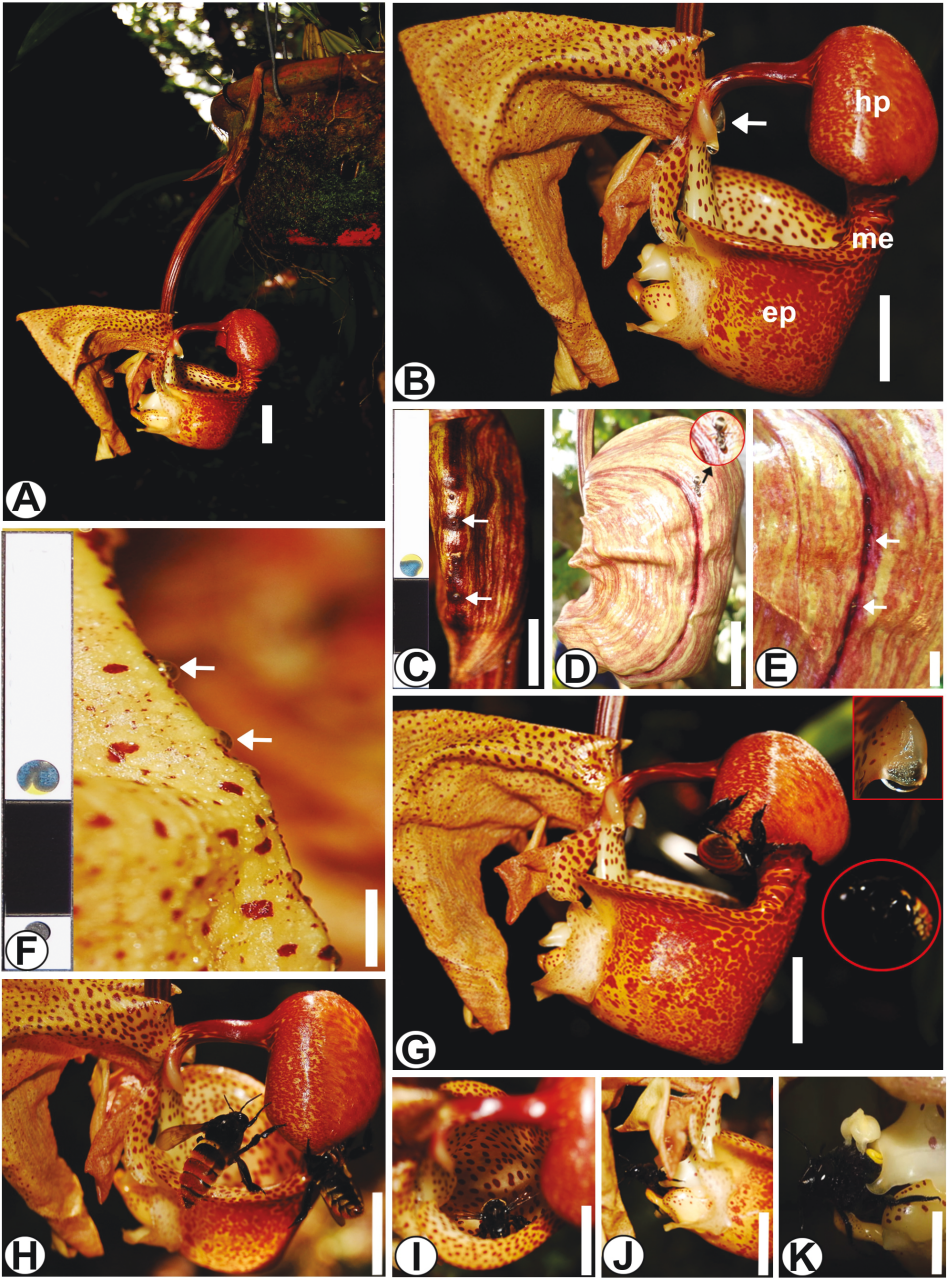
Habit and morphology of *Coryanthes macrantha*. (A) Habit and pendant inflorescence. (B) Lateral view of flower, note pleuridias (arrow). (C) Bracts, note nectaries (arrow). (D) Ant foraging next of nectaries in floral bud (arrow). (E) Nectaries in veins of floral bud (arrow). (F) Sepal, in detail the exudate of the nectaries (arrow). (G) Presence of bees *Euglossini* in the hypochile of the labellum and detail of pleuridia in upper right part. (H) Stunned bees next to the hypochile. (I) Bee inside of the epichile. (J) Bee finding the exit of the lower region of the epichile. (K) Bee coming out of the epichile and removing the pollinator from the base of the column. Scale bars: A, B, D, G–J = 2 cm; C, K = 1 cm; E, F = 5 mm. Key to all figures: abs, abaxial surface; ads, adaxial surface; ch, chloroplast; cw, cell wall; d, dictyosomes; is, intercellular space; ep, epichile; hp, hypochile; m, microorganisms; me, mesochile; mi, mitochondria; ne, nectar secretion; nu, nucleus; rer, rough endoplasmic reticula; pl, plastids; va, vacuole; ve, vesicles.

The inflorescence is pendant and lateral (Fig. 1A), arising at the base of the pseudobulb, bearing 1–5 flowers. The bracts are ovate. The flowers with ovary together with the pedicel are up to 17 cm long, slightly yellowish with reddish macules and very fragrant. The sepals are free from each other, the dorsal sepal is positioned parallel to the column with a fully reflex apex and the sides are patent to the column with strongly revolved margins (Fig. 1B). The petals are narrower than the sepals, with wavy margins, almost always overhanging the ovary. The labellum is the most prominent structure of the flower and is divided into three segments (Fig. 1B): the subsquadrate epichile, with a deep cavity in lateral view, stores an aqueous solution secreted by the horns (pleuridia); the mesochile, which is semi-tubular, externally and internally vinaceous, fleshy and externally pubescent in the central region; and the hypochile, which is orange-red and slightly hairy. The spine is subtubular, with an enlarged apex with two pleuridia that secrete a fluid exudate that accumulates in the epichile, a structure similar to a ‘bucket’ (Fig. 1B). The two pollinia are yellow with a rounded viscidium.

Ants (*Azteca* sp.) foraged in the bracts (Fig. 1C) and sepals (Fig. 1D–F) region in both pre-anthesis and post-anthesis. Analysis of the secretion with Glycophyte Plus (>25.0 mmol L^−1^) was positive in bract and sepal nectaries at both stages (Fig. 1C and F).

Anthesis occurred at approximately 0700 h, with simultaneous emission of fragrances. Five minutes later, male euglossine bees (*Eulaema* sp.) landed on the adaxial surface of the hypochile and foraged on the abaxial surface where the osmophores are located (Fig. 1G and H). The bee, during foraging, falls into the epichile (bucket) (Fig. 1I), which accumulates an aqueous substance secreted by the horns (pleuridia). Prevented from flying due to the slippery wall of the epichile, the bee after a few seconds finds the exit located in the inferior region of the epichile at the end of the column (Fig. 1J), where the viscidium is located. This allows the gluing of the pollinary onto the bee’s scutellum (Fig. 1K).

**Figure 2.**
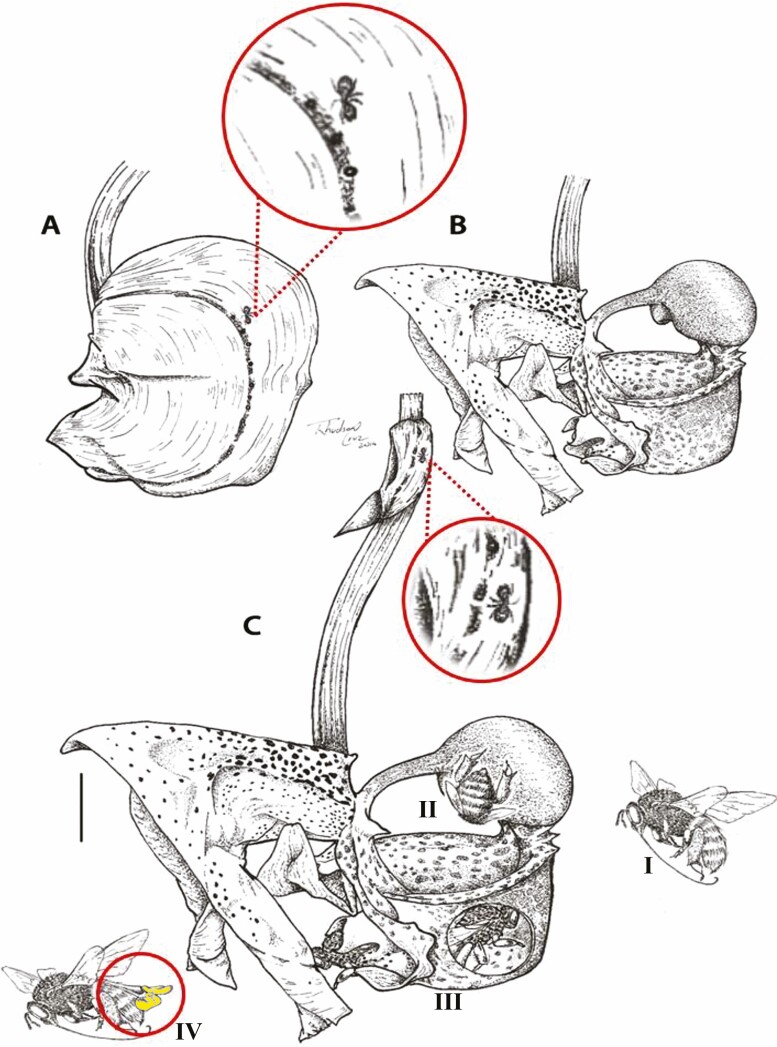
Foraging of visitors in *Coryanthes macrantha*. (A–C) Ant *Azteca* sp., in sepals and bracts before and after anthesis. (B) Flower in anthesis (C—Steps I–IV): I—Landing of the bee *Euglossini*, *Eulaema* sp., on the labellum (hypochile); II—The bee entering the excavated hypochile and collecting the oil; III—The bee inside the epichile and its exit from the side; IV—Polinarian being taken over the scutellum of the bee. Scale bars: 2 cm. For key, see Fig. 1.

A simplified scheme demonstrates the sequence of interactions between *C. macrantha* flowers, ants and bees. Ants forage in the sepal region of the flower bud and the flower bracts (Fig. 2A–C). Five minutes after anthesis, the bees that carried out the pollination process described above, at the same time with the ant’s presences (Fig. 2C—Steps I–IV).

### Anatomy of bract, labellum, sepals and pleuridia

#### Bract.

**Figure 3.**
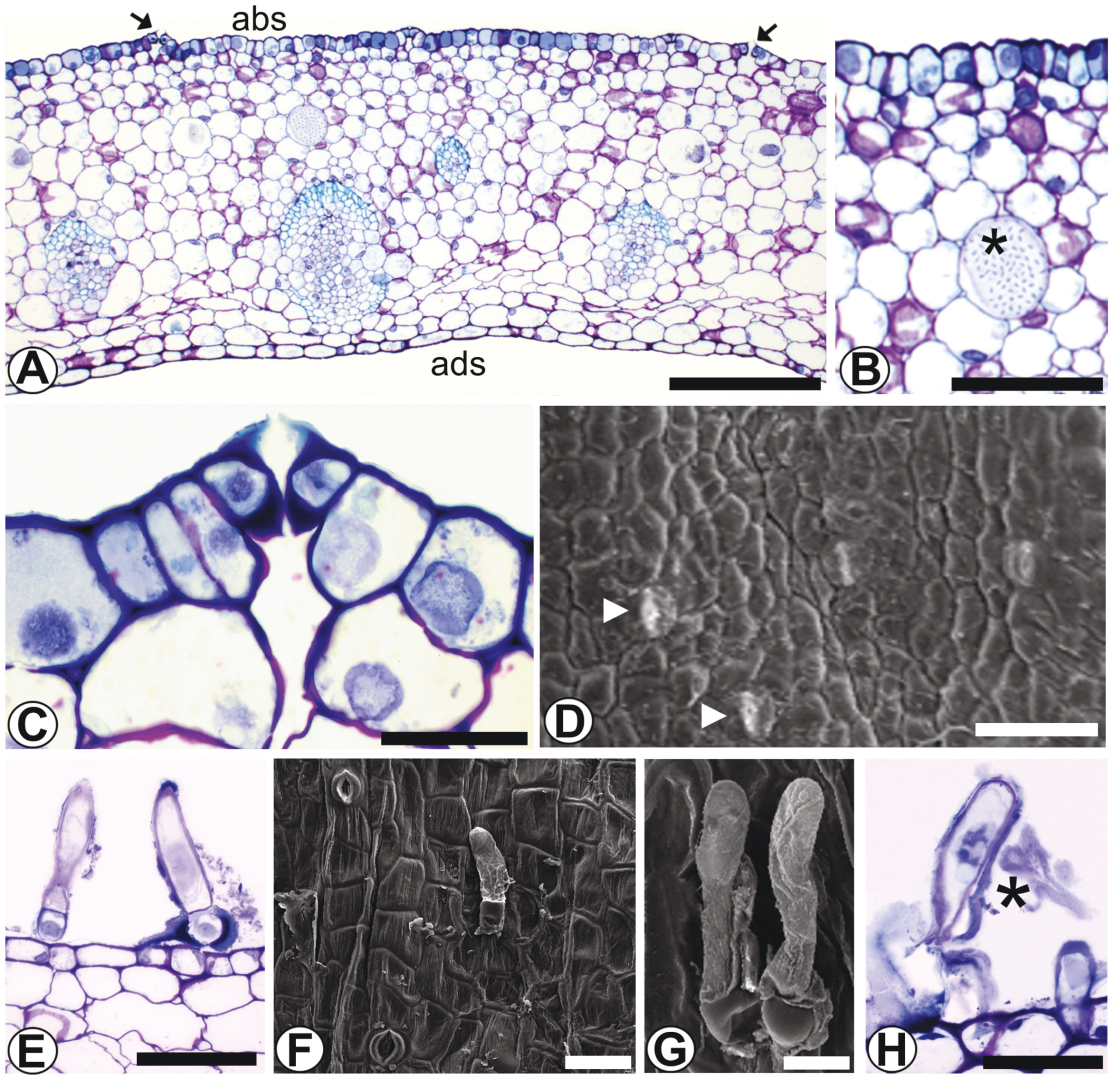
Bracts of *Coryanthes macrantha.* (A) Cross-section of bract, nectariferous stomata (arrow). (B) Cristaliferous idioblasts in the mesophyll (*). (C) Nectariferous stomata. (D) Nectariferous stomata (arrowhead) on the bract surface (SEM). (E–H) Trichomes digitiform (colleter) on the adaxial surface. (F and G) Trichomes digitiform (colleter) (SEM). Scale bars: D = 130 μm; A = 100 μm; B = 50 μm; E–G = 20 μm; C, H = 10 μm. For key, see Fig. 1.

Two types of secretory structures, nectaries and colleters, were identified in the bracts (Fig. 3A and E).

Nectaries have nectariferous stomata for nectar secretion, located in the abaxial region (Fig. 3A). These stomata are elevated slightly above the other epidermal cells in this region (Fig. 3C). Adjacent to this region of the epidermis is the nectariferous parenchyma. The mesophyll is composed of a layer of subepidermal nectariferous parenchyma (Fig. 3A and B) and below this, the fundamental parenchyma is associated contains collateral vascular bundles (Fig. 3A).

Colleters have deciduous digitiform trichomes located on the abaxial surface (Fig. 3E–H). Finger-shaped trichomes on the adaxial surface are also seen, but they are not colleters.

#### Sepals.

**Figure 4.**
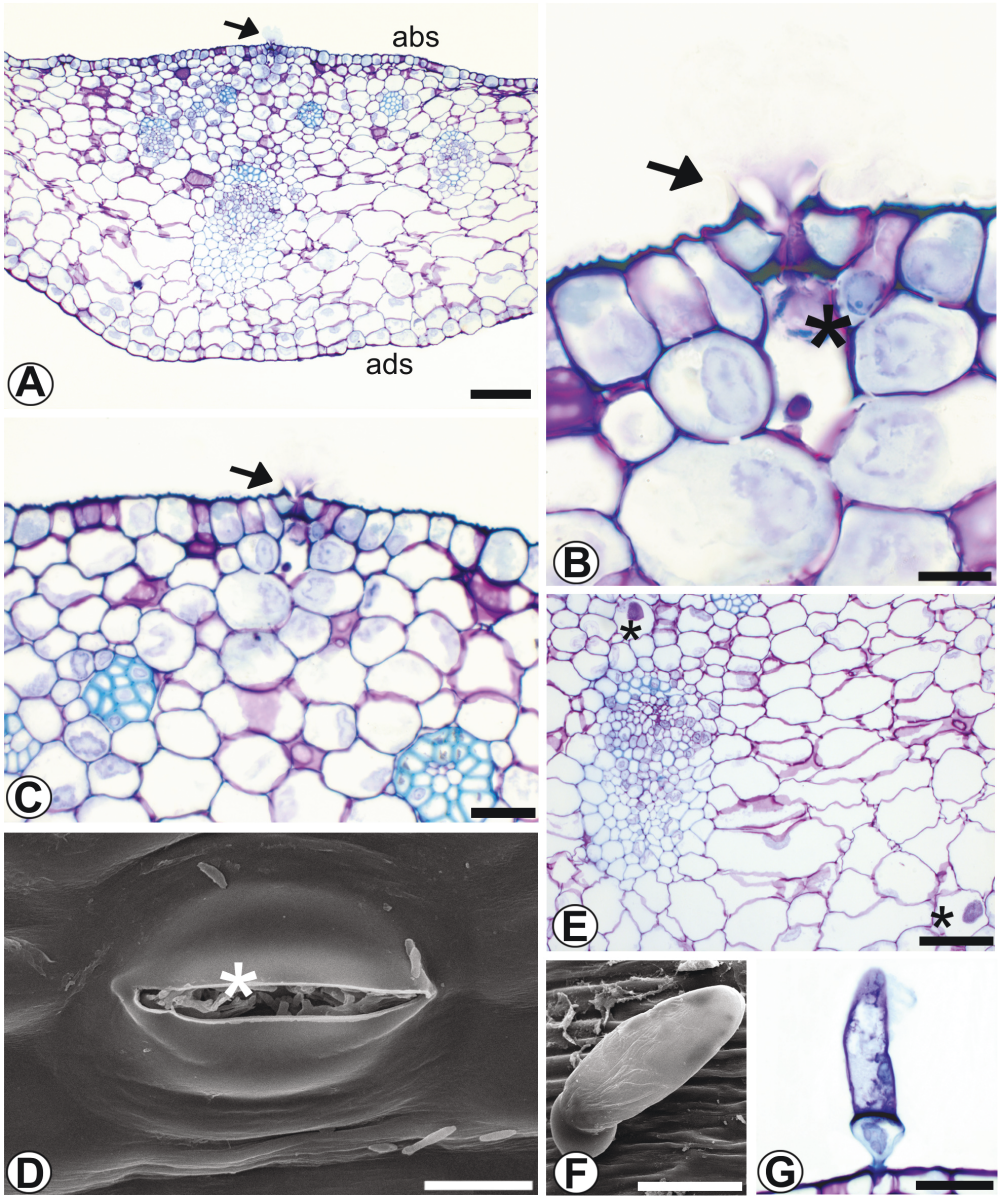
Sepal of *Coryanthes macrantha*. (A) Cross-section of sepal, nectariferous stomata (arrow). (B) Evident secretion of nectar (arrow), nectar in the substomatal air space (*). (C) Mesophyll, nectariferous parenchyma, and subnectary parenchyma, secretion of nectar (arrow). (D) Fungal colonization, which has grown on the nectar (stomata). (E) Collateral vascular bundle, cristaliferous idioblasts (*). (F and G) Digitiform trichome in the adaxial surface, (F) (SEM). Scale bars: A = 100 μm; E = 50 μm; F = 40 μm; C–G = 20 μm; B, D = 10 μm. For key, see Fig. 1.

In transverse view, the epidermis of the abaxial and adaxial faces of the sepals is unistratified and anisodiametric, with a thin cuticle (Fig. 4A). Cells of the adaxial face have sinuous outer anticlinal walls and straight to gently curved inner walls; cells of the abaxial face have straight to gently curved inner and outer periclinal walls. The mesophyll has 1–2 layers of subepidermal nectariferous parenchyma and approximately four layers of subnectariferous parenchyma with anisodiametric cells and collateral vascular bundles. The nectariferous stomata on the adaxial face of the post-anthesis sepal were analysed (Fig. 4B–D). Crystalliferous idioblasts (raphides) are present, randomly distributed in the fundamental parenchyma (Fig. 4E). The sepals are epistomatic and have deciduous digitiform trichomes only on the adaxial surface (Fig. 4F and G). The stomata, similarly to the ones in the bracts, are elevated slightly above the epidermis.

#### Labellum.

**Figure 5.**
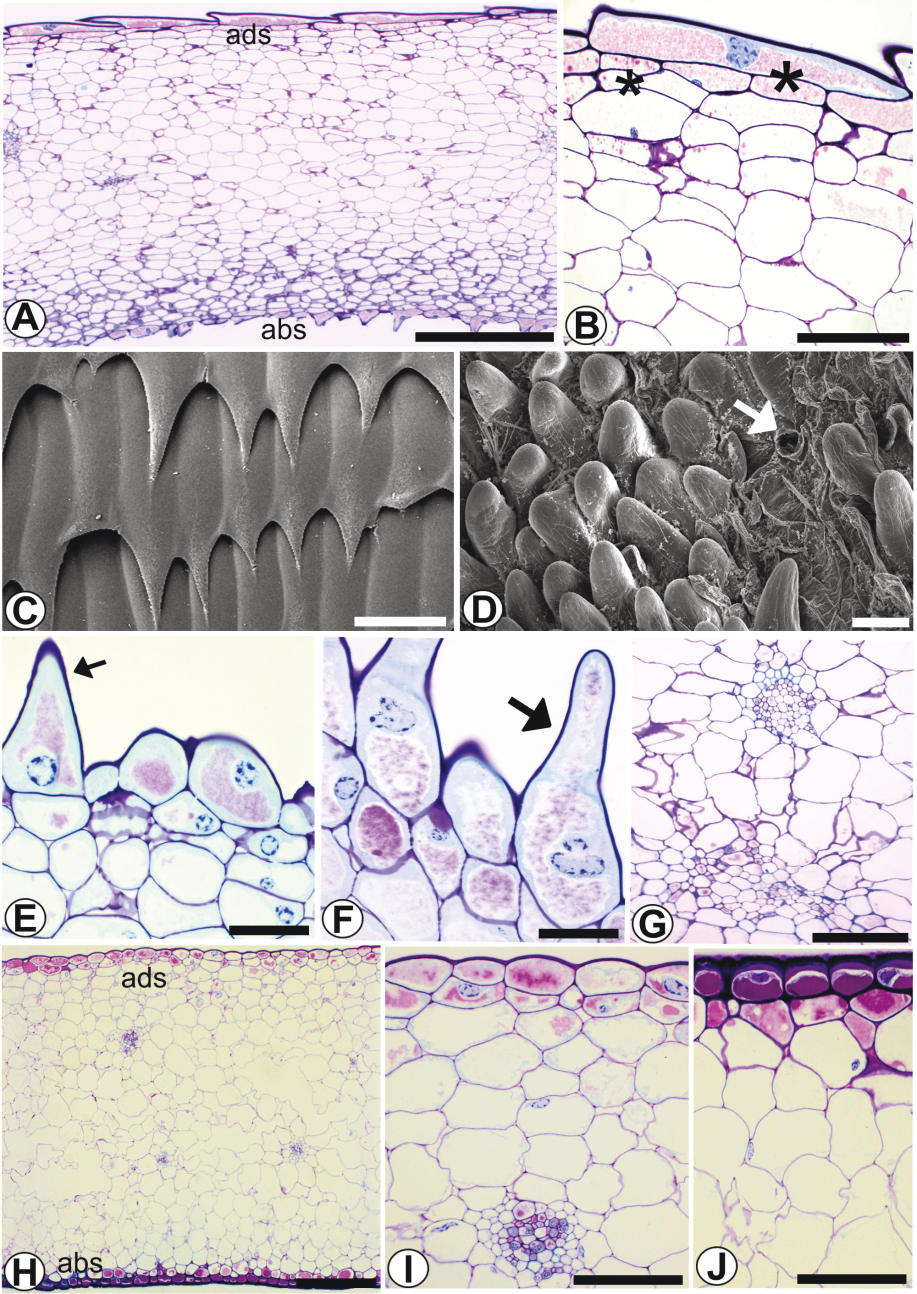
The labellum of *Coryanthes macrantha*. (A) Cross-section of the labellum (hypochile). (B) Detail of the epidermal cell with the format of the squama, note lipophilic contents (*). (C) The epidermis and your cells with the format of the squama (SEM). (D) Papillary cells of epidermis on the abaxial surface (SEM), stomata (arrow). (E and F) Papillary cells of different sizes. (G) Collateral vascular bundle. (H) Cross-section of the labellum (epichile). (I) The adaxial face of epichile and collateral vascular bundle in the parenchyma. (J) The abaxial face of epichile. Scale bars: A = 500 μm; H = 200 μm; G = 100 μm; D = 60 μm; B, I, J = 50 μm; C, E, F = 20 μm. For key, see Fig. 1.

On the abaxial face of the labellum (hypochile) are the epidermal-type osmophores. In cross-section, the hypochile has different epidermal cells (Fig. 5A). The epidermis of the adaxial surface has cells with an elongated outer periclinal wall with a scale shape and cellular content (Fig. 5B and C). The epidermis of the abaxial surface, the osmophore, has papillary, unicellular, slightly wrinkled cells of different sizes (Fig. 5D–F). In the underlying parenchyma the vascular bundles are collateral (Fig. 5G). In transverse section, the epichile has similar epidermal cells on the adaxial and abaxial faces, slightly rounded to flattened (Fig. 5H). The epidermal cells on both faces have an acidic mucilaginous content (Fig. 5I and J). The vascular bundle is collateral (Fig. 5I).

#### Pleuridia.

**Figure 6.**
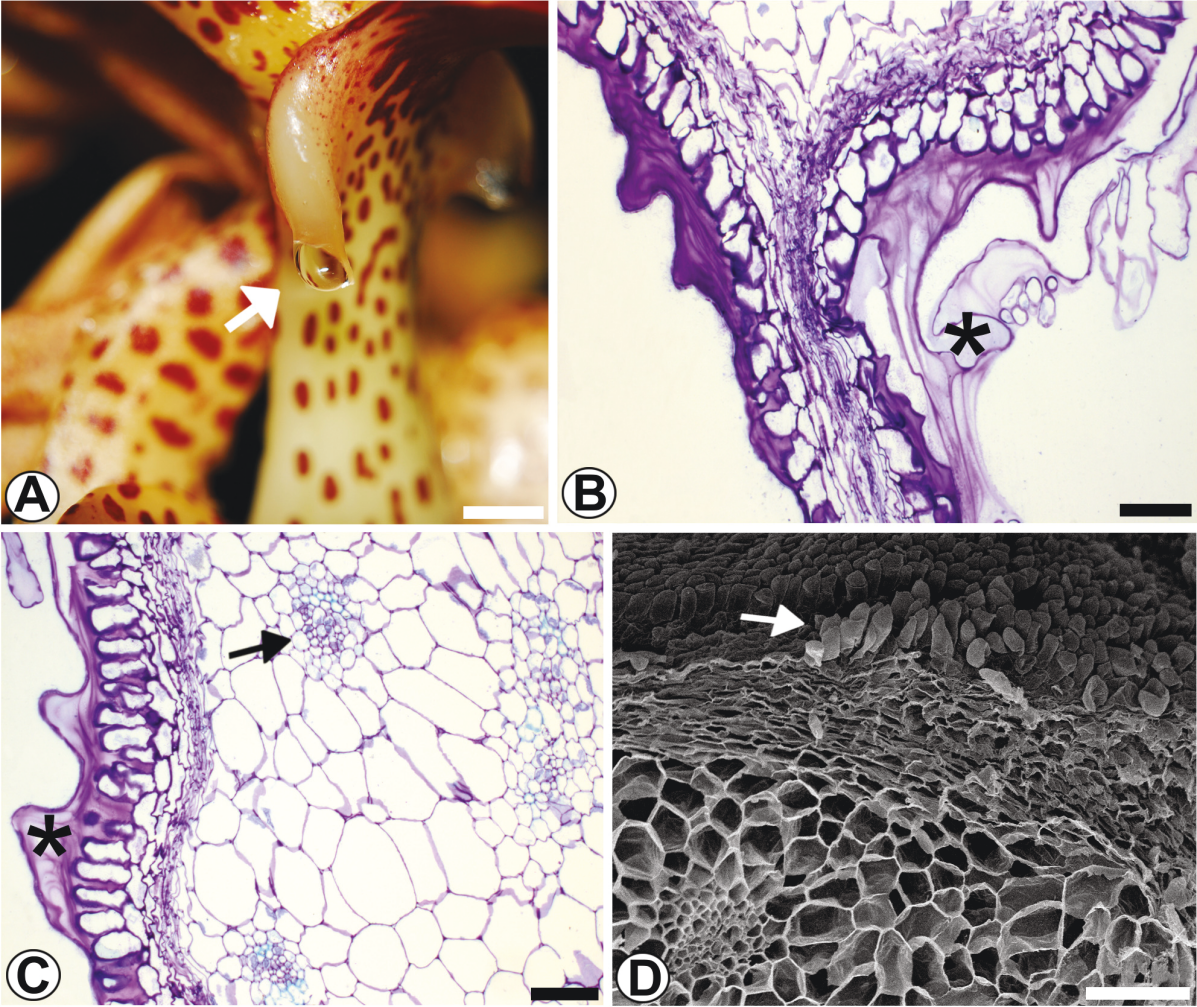
Morphology of pleuridias in *Coryanthes macrantha*. (A) Secretion drip in the epichile. (B) Longitudinal section of pleuridia, mucilaginous content (*). (C) Cross-section of pleuridia, mucilaginous content (*). (D) Papillary cells (arrow) and parenchyma of pleuridia (SEM). Scale bars: A = 2 cm; D = 200 μm; B, C = 100 μm. For key, see Fig. 1.

At the apex of the column are the two horns called pleuridia (Fig. 6A). The pleuridia are large, rectangular, slightly falcate and secrete a viscous fluid (mucilage). The epidermis in longitudinal (Fig. 6B) and transverse (Fig. 6C) views has a unistratified, anisodiametric surface, a thick cuticle and a voluminous subcuticular space between the epidermal cells (Fig. 6D). The epidermal cells are papillary, without stomata; in the cortical region are randomly distributed collateral vascular bundles.

#### Histochemistry.

**Figure 7.**
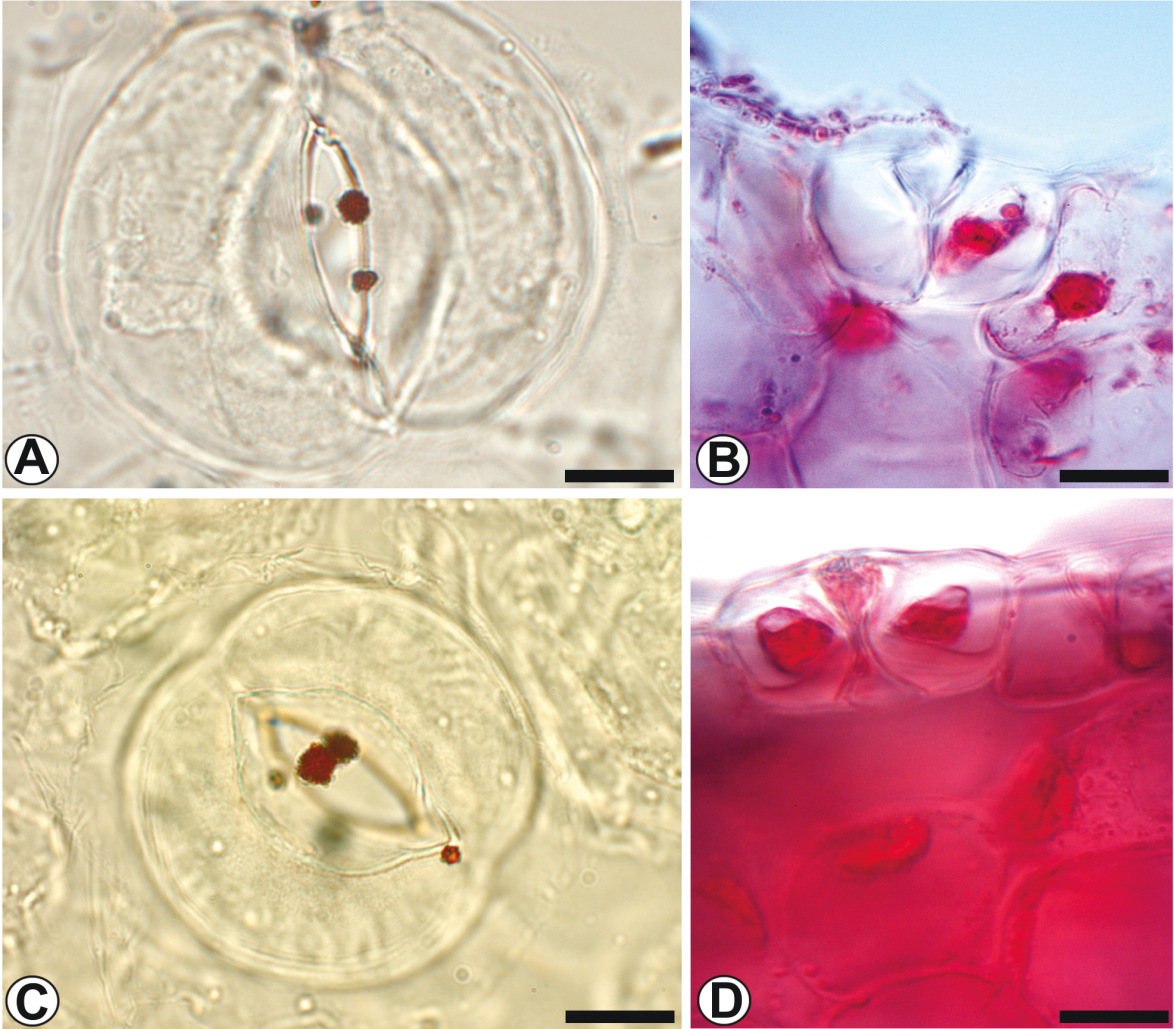
Histochemical analysis of *Coryanthes macrantha* of the median regions of bracts and sepals (cross-section). (A and B) Bract. (C and D) Sepal. (A, C) Fehling reagent. (B, D) Staining with Xylidine revealed the presence of protein bodies in the nectaries. Scale bars: A–D = 10 μm. For key, see Fig. 1.

**Figure 8.**
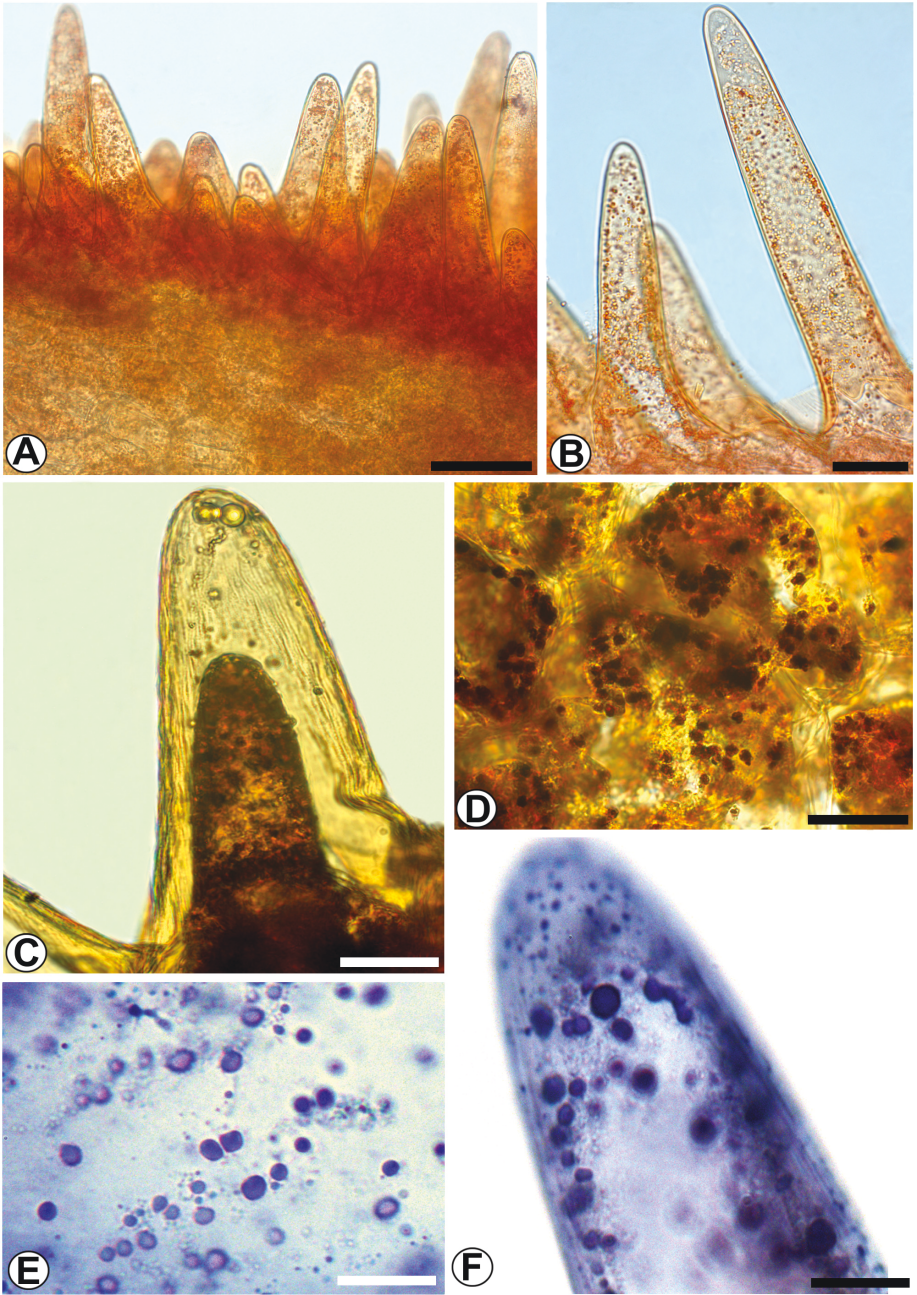
Histochemical analysis of the abaxial and adaxial surface of the labellum (hypochile) of *Coryanthes macrantha*. (A and B) Staining with Sudan III in the papillose cells, notes the presence of lipid bodies. (C and D) The distribution of starch grains (Lugol reagent) in the secretory papillose cells and subsecretory parenchyma. (E and F) Essential oils presence by NADI reagent. Scale bars: A = 50 μm; B = 20 μm; C–F = 10 μm. For key, see Fig. 1.

**Figure 9.**
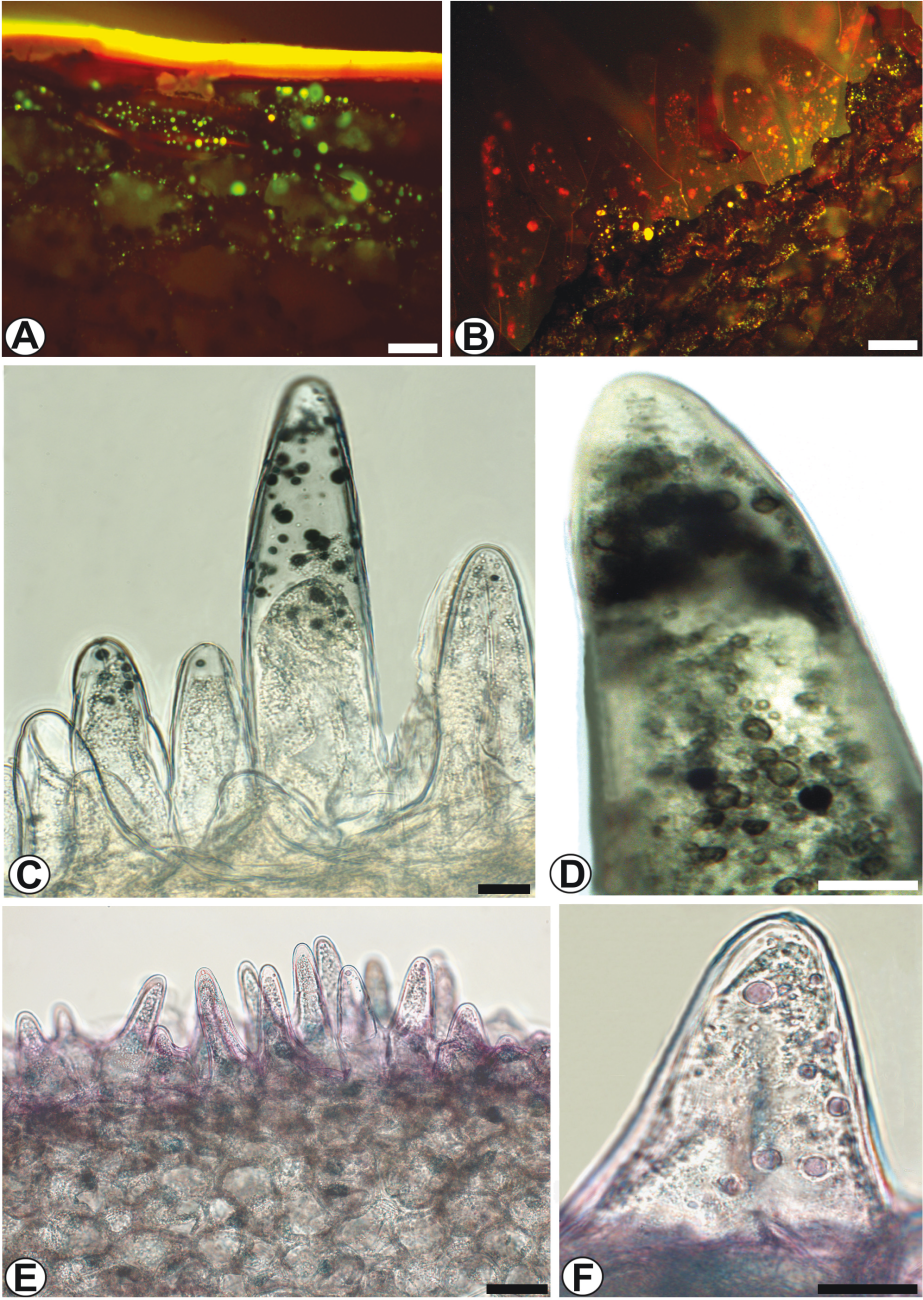
Histochemical analysis of cross-section of the abaxial and adaxial surfaces of the labellum (hypochile) of the *Coryanthes macrantha*. (A and B) Fresh material of labellum with Neutral red in UV light. (C and D) Staining bodies with fatty acids (copper acetate/rubeanic acid) in the papillose cells. (E and F) Bodies of neutral lipids (Nile blue sulfate). Scale bars: A, B, E = 50 μm; C = 20 μm; D, F = 10 μm. For key, see Fig. 1.

Histochemical analysis of *C. macrantha* in both bract and sepal nectaries revealed the following compounds: monosaccharides (reducing sugars) (Fig. 7A and C) and total proteins (Fig. 7B and D). In the region of the labellum (adaxial and abaxial face), the osmophore (hypochile), the tests showed: total lipids (Fig. 8A and B); starch (Fig. 8C and D); terpenoids (essential oils) (Fig. 8E and F); total lipids in UV light (Fig. 9A and B); fatty acids (Fig. 9C and D); and acidic and neutral lipids (Fig. 9E and F). Bract, sepal and labellum test results are summarized in Table S1 in the Supporting Information.

#### Phytochemicals.

The components identified in the floral fragrance of *C. macrantha* (Pa – 175) in percentages above 5 % were: *para*-anisyl acetate, *n*-hexanal, deca-(2E,4E)-dienal, 2-pentyl-furan and (Z)-β-farnesene (Table 1).

**Table 1.** Analysis of the floral fragrance of *Coryanthes macrantha* (Pa – 175).

RI	Constituents	Characterization	%
1345	Dihydroisojasmone	Acetone	0.16
798	***n*-Hexanal**	**Aldehyde**	**29.45**
1104	Nonanal	Aldehyde	2.07
1295	Deca-(2E,4Z)-dienal	Aldehyde	1.23
1317	**Deca-(2E,4E)-dienal**	**Aldehyde**	**6.33**
1256	*para*-Anisaldehyde	Benzaldehyde	3.07
1360	Eugenol	Carboxylic acid	0.13
1407	Methyl-eugenol	Carboxylic acid	0.88
1422	***para*-Anisyl acetate**	**Carboxylic acid**	**10.6**
1600	1-[2-(Isobutyryloxy)-1-methylethyl]-2,2-dimethylpropyl 2-methylpropanoate	Ester	0.66
2097	Methyl linoleate	Fatty acid	0.34
1376	*n*-Undecanol	Fatty alcohols	0.53
1437	Methyl undecanoate	Fatty esters	0.4
1002	**2-pentyl-Furan**	**Furane**	**5.66**
1471	β-Acoradiene	Hydrocarbon	0.19
1640	α-Acorenol	Hydrocarbon	0.56
2099	*n*-Heneicosane	Hydrocarbon	0.17
2311	*n*-Tricosane	Hydrocarbon	0.52
2401	*n*-Tetracosane	Hydrocarbon	0.09
1291	Safrole	Hydrocarbon	0.31
1418	*cis*-α-Bergamotene	Hydrocarbon	0.23
1439	*trans*-α-Bergamotene	Hydrocarbon	2.65
1446	**(Z)-β-Farnesene**	**Sesquiterpene**	**10.83**
1459	(E)-β-Farnesene	Sesquiterpene	1.01
1482	γ-Curcumene	Sesquiterpene	1.63
1485	α-Curcumene	Sesquiterpene	0.75
1508	(E,E)-α-Farnesene	Sesquiterpene	0.31
1511	β-Bisabolene	Sesquiterpene	0.64
1515	β-Curcumene	Sesquiterpene	3.13
1520	(Z)-γ-Bisabolene	Sesquiterpene	0.15
1527	β-Sesquiphellandrene	Sesquiterpene	0.4
1547	*cis*-Sesquisabinene hydrate	Sesquiterpene	1.2
1584	*trans*-Sesquisabinene hydrate	Sesquiterpene	2.2
1676	*epi*-β-Bisabolol	Sesquiterpene	2.32
1689	α-Bisabolol	Sesquiterpene	0.17
1392	7-*epi*-Sesquithujene	Terpene	2.5
1455	Geranylacetone	Terpene	0.12
1490	(E)-β-Ionone	Terpene	1.16
1612	β-Atlantol	Terpene	0.32

Bold indicates emphasis on the class of chemical compounds analysed. RI = retention index. PA is the code of the sample in the laboratory at Museu Paraense Emílio Goeldi.

### Transmission electron microscopy

#### Bract nectar.

**Figure 10.**
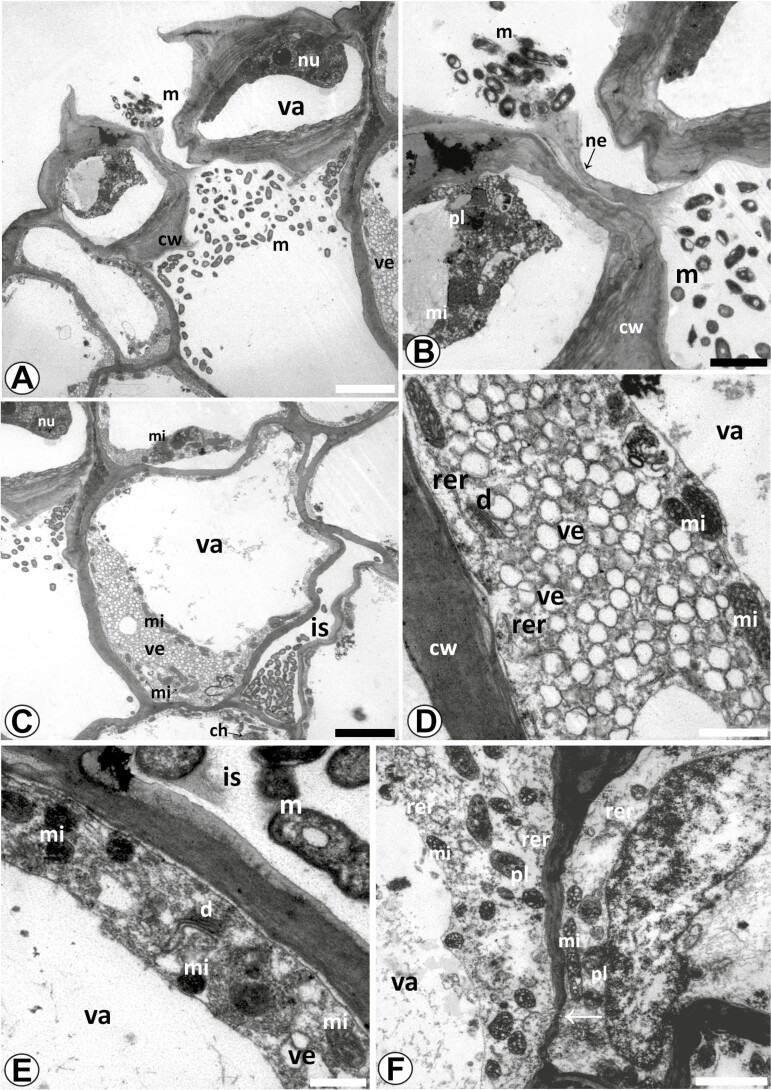
Analysis using TEM of nectaries in the bracts of *Coryanthes macrantha*. (A) Nectariferous stomata in the abaxial surface of the bracts, note the presence of eukaryotic microorganisms in the substomatal chamber. (B) Microorganism in the secretion of nectar. (C) Cells of nectary parenchyma with evident vacuole, numerous vesicles, mitochondria and large intercellular spaces. (D) Rough endoplasmic reticula and dictyosomes have also been observed in these cells of the nectary parenchyma. (E and F) Cells of subnectary parenchyma containing huge vacuolated, numerous vesicles, mitochondria, rough endoplasmic reticula, plastids and dictyosomes. (F) Detail of eukaryotic microorganisms in the intercellular spaces. (F) Plasmodesmata (arrow) have also been observed in these cells of the subnectary parenchyma. Scale bars: A, C = 5 μm; B, F = 2 μm; D = 1 μm; E = 0.5 μm. For key, see Fig. 1.

The bract nectary analysed with TEM was collected 1 day before floral anthesis. Guard cells of the nectary stomata have thickened walls, large nuclei, a dense nucleolus and large vacuoles (Fig. 10A), the same cytological characteristics observed in light microscopy. However, in TEM, eukaryotic microorganisms were observed in the nectar secretion and the substomatic chamber space (Fig. 10B).

The cells of the nectariferous parenchyma have numerous vesicles, probably originating from dictyosomes and/or endoplasmic reticulum (Fig. 10D), with abundant mitochondria, a well-developed vacuole and, rarely, chloroplasts (Fig. 10C), in addition to ribosomes, rough endoplasmic reticulum and active dictyosomes (Fig. 10D). No amyloplasts were observed. In the vicinity of the periplasmic space, mitochondria, rough endoplasmic reticulum and dictyosomes were observed (Fig. 10D).

In cells of the subnectariferous parenchyma, the cytoplasm contains well-developed vacuoles, mitochondria, dictyosomes, vesicles, abundant rough endoplasmic reticulum and plastids containing small starch grains (Fig. 10E and F). Eukaryotic microorganisms were observed in the large intercellular spaces of this layer of cells (Fig. 10E). Plasmodesms were also seen crossing the walls between cells (Fig. 10F).

## Discussion

### Ecological interactions

Members of *Coryanthes* are epiphytic herbs that have always aroused the curiosity of great naturalists such as Darwin, mainly due to the peculiar morphology of their flowers, the dripping of pleuridia and their ingenious pollination mechanism (Darwin 1882; Gerlach 2011).

In this study, *Azteca* ants were observed interacting with *C. macrantha* nectaries. A similar interaction with ants occurs in other species of Orchidaceae, such as in *Epidendrum denticulatum*, *Rodriguezia venusta* and in species of *Cohniella*. Ants forage in extrafloral nectaries without interfering with the behaviour of pollinators, protecting reproductive structures and thus helping to increase the probability of successful pollination as well as plant development (Almeida and Figueiredo 2003; Leitão *et al.* 2014; Kettler *et al.* 2019).

In addition to extrafloral and floral nectaries, osmophores are present in the hypochile of the abaxial face of the labellum of *C. macrantha*. The osmophores or scent glands are the main secretory structures of species of Stanhopeinae (Vogel 1962, 1990; Curry *et al*. 1991; Antoń *et al.* 2012; Casique *et al*. 2018). These structures provide volatile compounds as a reward for their exclusive pollinators, male euglossine bees (Gerlach 2003; Antoń *et al*. 2012; Pansarin *et al.* 2014; Adachi *et al.* 2015).

The flowers of *C. macrantha* are as complex as the flowers of *Gongora* and *Stanhopea* (Dodson *et al*. 1969; Gerlach and Schill 1989; Nunes *et al.* 2017). This complexity is due to the morphology of the labellum of these Stanhopeinae genera, which have pollination processes considered to be the most ingenious in the plant kingdom (Dodson *et al*. 1969; Dressler 1993). The pollination mechanism in the genera *Gongora*, and *Stanhopea* involves only the directed fall of the pollinator onto the column holding the pollinia (Adachi *et al*. 2015; Nunes *et al.* 2017; Casique *et al*. 2021). In *Coryanthes* the mechanism is more complex, the male euglossine bees of appropriate size are attracted by specific odours and they fall into the epichile, which is filled with a liquid secreted by the pleuridia. The bee can leave only through a small lateral tunnel formed by the base of the column and the labellum, thus removing the pollinator (Nazarov and Gerlach 1997; Gerlach 2011).

### Elaiophores, nectaries and pleuridia

Odour and nectar are important in maintaining the plant–pollinator relationship (Vogel 1969; Melo*et al.* 2010). Floral odour is primarily responsible for attracting pollinators from long distances, while nectar is one of the main floral rewards of plants (Proctor *et al.* 1996). Floral perfume is produced by osmophores (smell glands) that occur in a large group of plants, for example in Araceae, Orchidaceae, Apocynaceae (Asclepiadoideae) and Burmanniaceae (Vogel 1990; Dressler 1993). In Orchidaceae, osmophores are found almost exclusively in the labellum (Vogel 1990). In other species of orchids, the source of the floral aroma may be located at the tips of the petals and in the ovaries (Esau 1965; Swanson *et al.* 1980; Silveira 2002; Kowalkowska *et al.* 2015).

Osmophores in flowers have a diverse histological structure (Vogel 1990). The epidermal surface of osmophores may have different morphological characteristics, i.e. glabrous cells with simple cubical epidermal secretory cells (Vogel 1990; Curry *et al*. 1991; Kettler *et al*. 2019; Tölke *et al*. 2019), or may have papillae and/or multi- or unicellular trichomes (Curry *et al*. 1991; Davies and Turner 2004; Naczk *et al*. 2018). In addition, osmophores may have an epidermis with a striated to smooth cuticle and subepidermal secretory parenchyma cells (Vogel 1990; Tölke *et al*. 2019).

The cellular characteristics described here for the epidermal osmophore of *C. macrantha* are similar to those previously described for other species of Stanhopeinae, such as *Gongora bufonia*, *S. grandiflora*, *Stanhopea graveolens*, *Stanhopea oculata*, *Stanhopea wardii*, *Sievekingia* and *Cirrhaea* (Stern *et al.* 1987; Curry *et al*. 1991; Antoń *et al*. 2012; Pansarin *et al*. 2014; Adachi *et al*. 2015; Casique *et al*. 2018), which have glabrous epidermal osmophores.

Nectaries are specialized glands in plant tissue that secrete nectar, which is comprised mainly of monosaccharides, disaccharides, proteins, amino acids, water and other compounds (Fahn 1979; Elias 1983; Endress 1994). Nectaries occur in several families of angiosperms and gymnosperms, and in certain species of ferns (Endress 1994; Pacini *et al.* 2003). The main function of nectaries is to attract visitors, both pollinators (nuptial nectaries) and defenders (extranuptial nectaries) such as ants (Pacini and Nepi 2007; Heil 2011). Recent studies have shown that nectaries can also function as a defence against microbial invasion, due to the presence of proteins in the nectar (Park and Thornburg 2009; Harper *et al*. 2010; Zhou *et al*. 2016).

Extrafloral nectaries of *C. macrantha* were observed in bracts. These are commonly associated with plant defence, as they attract invertebrate predators, usually ants, whose presence and activity can reduce herbivory (Heil and McKey 2003; Bronstein *et al.* 2006; Gerlach 2011).

Nectaries vary in location and morphology but have similar anatomical features (Tölke *et al*. 2019). They consist of a nectariferous epidermis, with or without trichomes and stomata involved in secretion, composed of nectariferous parenchyma and subnectariferous parenchyma (Fahn 1988; Nepi 2007; Escalante-Pérez and Heil 2012). Nectaries may be connected to the phloem, the xylem, to both, or may lack a direct vascular connection (Fahn 1988). Nectar from the bract and sepal of *C. macrantha* is exuded through stomata. The stomata involved in nectar secretion are sometimes termed nectarostomes (Smets and Cresens 1988). When nectar is exuded by nectariferous stomata, these may remain permanently open (Heil 2011).

In Orchidaceae, nectar exudation by nectariferous stomata is common in *Maxillaria anceps* (Davies *et al.* 2005) and species of *Disa* (Johnson *et al.* 1998; Hobbhahn *et al*. 2013; Johnson *et al.* 2013) and *Cohniella* (Kettler *et al*. 2019). Ultrastructural analyses of the nectariferous parenchyma of *C. macrantha* bracts revealed characteristics similar to those of other orchid species, including a dense cytoplasm, numerous mitochondria, dictyosomes, numerous vesicles and rough endoplasmic reticulum, organelles related to nectar synthesis in orchids (Figueiredo and Pais 1992; Stpiczyńaska and Matusiewicz 2001; Davies *et al*. 2005; Melo *et al*. 2010; Swiczkowska and Kowalkowska 2015) as well as in other plants (Gaffal *et al.* 1998; Nepi 2007; Vassilyev 2010; Tölke *et al*. 2019).

The nectary of the bract of *C. macrantha* was collected at the time of its secretory activity. Transmission electron microscopy observation showed that the cells of the nectariferous parenchyma contained relatively large vacuoles. While nectar secretion is taking place, the vacuoles of nectariferous parenchyma cells tend to increase in volume (Nepi 2007), the cytoplasm is dense and rich in ribosomes and mitochondria, the rough endoplasmic reticulum is highly developed and the dictyosomes are active by the numerous vesicles of this organelle. Plastids sometimes contain starch grains (Escalante-Pérez and Heil 2012; Tölke *et al*. 2019). In most of the nectaries described previously, during the secretory phase the energy provided by the starch grains to the nectary is rapidly consumed (Stpiczyńska *et al.* 2005a, b; Nepi 2007; Pacini and Nepi 2007; Paiva and Machado 2008; Heil 2011). In the course of the secretory process this reserve tends to decrease (Escalante-Pérez and Heil 2012). This may be the reason that starch grains were not observed in these cells of the nectariferous parenchyma of the *C. macrantha* bract.

The larger intercellular spaces that are close to the substomatic chambers may be an anatomical adaptation that facilitates nectar transport through the apoplast (Nepi 2007; Leitão *et al*. 2014). The mode of secretion of nectar from *C. macrantha* bracts is granulocrine, in which pre-nectar is transported in vesicles that may move through a symplastic pathway, secreted via exocytosis, and an apoplastic pathway (Vassilyev 2010; Heil 2011; Abedini *et al*. 2013; Tölke *et al*. 2019). The floral and extrafloral nectaries described in the bracts and sepals of *C. macrantha* (Gerlach 2011) are here confirmed and anatomically detailed for the first time for this genus.

Idioblasts containing raphides were also observed in the bracts and sepals of *C. macrantha*, a common characteristic in leaves of Stanhopeinae species (Stern 2014). Idioblasts are also common in tepals of other orchid species (Stpiczyńska *et al*. 2003; Kowalkowska and Margońska 2009; Kowalkowska *et al*. 2015), and are often accompanied by secretory cells such as nectaries, resin glands and elaiophores (Stpiczyńska *et al.* 2011; Davies and Stpiczyńska 2012, 2019; Casique *et al*. 2018), which defend against herbivores (Prychid and Rudall 1999). Additionally, these calcium crystals may only be being stored, as they are present in excess in the cytosol (Paiva and Machado 2008).

The secretory epidermis of pleuridia of *C. macrantha* exudes a viscous mucilage. Similarly, Schnepf *et al*. (1983) ruled out the possibility that the corresponding secretion in *C. speciosa* is nectar.

### Chemical composition of the secretion of the osmophores of the labellum (hypochile)

The scent of orchid flowers is produced and exuded by osmophores or scent glands (Vogel 1990; Silva 1992; Adachi *et al*. 2015). Floral aromas are intimately involved in attracting euglossine bee pollinators (Dressler 1993; Hetherington-Rauth and Ramírez 2015). Based on the histochemical and chemical analyses performed in this study, the exudate of the labellum (hypochile) of *C. macrantha* is a mixed oil containing terpenoids, which are characteristic of osmophores, and compounds derived from high-molecular-weight fatty acids. These oils may help the bees ‘slip’ into the epichile, a possibility needing investigation.

A variety of floral aromatic compounds are known (Endress 1994). Their aromas may be pleasant, such as fatty-acid methyl esters, mono- and sesquiterpenes (aliphatic and cyclic), diterpenes, compounds with benzene rings and phenylpropanoids; or unpleasant, such as hydrocarbons, fatty acids and nitrogenous volatiles (ammonia, indole, cadaverine, putrescine) (Vogel 1983).

Flower aromas in Stanhopeinae species are composed of monoterpenes, sesquiterpenes and their derivatives, such as esters, ethers, indoles, lactones, epoxides and aldehydes, often in the form of aromatics (Gerlach 2009).

In species of *Stanhopea*, monoterpenes (α-pinene, β-pinene, sabinene, myrcene, limonene, eucalyptol (1,8-cineole) and ocimenes; Whitten and Williams 1992; Gerlach 2010) are commonly found. In *S. grandiflora*, compounds such as hexanal, limonene, nonanal, eugenol and octadeca-(3Z,13Z)-di-1-yl acetate were also identified and may be responsible for attracting pollinators (Casique *et al*. 2018). In *Stanhopea*, floral fragrances involve less-specialized compounds than in other genera of Stanhopeinae (Gerlach 2009).

Species of *Coryanthes* have different aromas. Phytochemical analyses have helped taxonomists to delimit the species, which in nature are pollinated by the same euglossine bees, despite the differences in colour or morphology of their flowers (Gerlach 2011).

Previous phytochemical studies identified the volatile components in *C. macrantha* flowers, in addition to alkaloids, seven chemotypes, one ester, ipsdienol/myristene B, 2-*N*-methylaminobenzaldehyde, undecatriene, 1,8-cineole, acimene and sesquiterpene (Gerlach and Schill 1993). Of these, 2-*N*-methylaminobenzaldehyde is considered rare in the plant kingdom (Gerlach 2009). In the present phytochemical analysis, this rare compound was not observed, possibly because of the environment where the plants were collected and observed.

In conclusion, the flowers of *C. macrantha* possess structures that secrete lipids and nectar, i.e. the osmophores and floral and extrafloral nectaries; the anatomical details of the nectaries are described here for the first time. In view of the diversity of these structures, it is important to analyse other species of *Coryanthes*, as well as other members of Stanhopeinae and Orchidaceae, to add anatomical data that will allow us to understand the floral evolution of the family.

## Supporting Information

The following additional information is available in the online version of this article—

Table S1. Histochemical analyses of exsudates of the secreting structures of *Coryanthes macrantha*.

Figure S1. Untreated sections and negative controls of tests performed on the labellum (hypochile—after treatment using lipid extractor solution) and bracts of *Coryanthes macrantha*. (A and B) Labellum untreated; (C) bract untreated; (D) negative control of Sudan III test on the labellum; (E) negative control of Nile blue sulfate test on the labellum.

Figure S2. Negative controls of tests performed on the labellum (hypochile—after treatment using lipid extractor solution) and bracts of *Coryanthes macrantha*. (A) Negative test of copper acetate/rubeanic acid on the labellum; (B) negative test of the NADI reagent on the labellum; (C and D) negative Xylidine Ponceae test of the bract after treatment with 10 % acetic anhydride solution in pyridine for 8 h.

plac039_suppl_Supplementary_Figure_S1

plac039_suppl_Supplementary_Figure_S2

plac039_suppl_Supplementary_Table_S1

## Data Availability

The original contributions presented in the study are included in the article/**Supporting Information**, further inquiries can be directed to the corresponding author.

## Notes

**Natural History and Conservation. Chief Editor:** F. Xavier Picó
