# Supplementary figures and images for: Coryanthes macrantha (Orchidaceae: Stanhopeinae) and their floral and extrafloral secretory structures: an anatomical and phytochemical approach
Source: AoB Plants. 2022 Sep 2;14(5):plac039. doi: 10.1093/aobpla/plac039 (PMC9525647; doi:10.1093/aobpla/plac039)

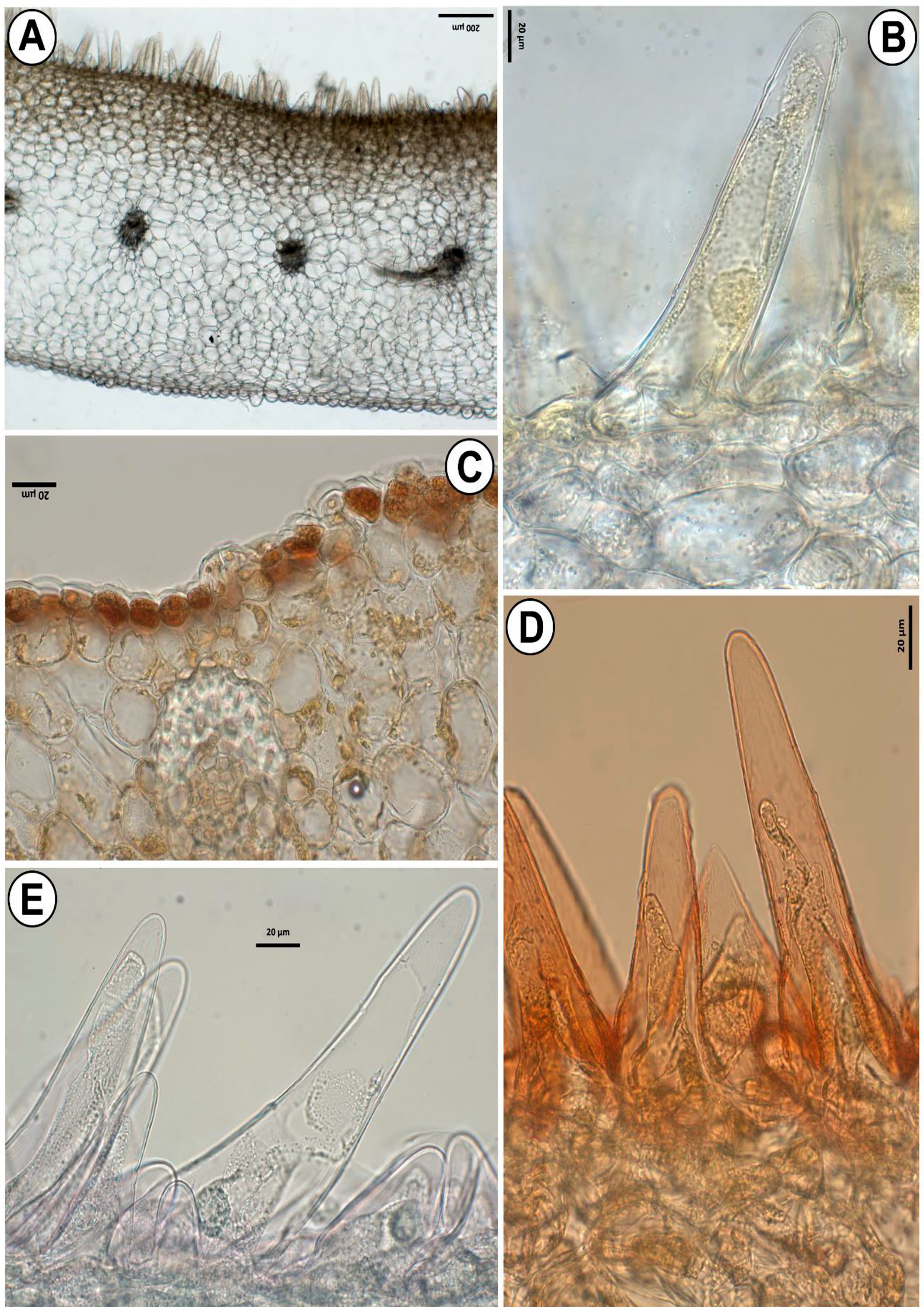

Supplement: plac039_suppl_Supplementary_Figure_S1 [file plac039_suppl_supplementary_figure_s1.jpeg]

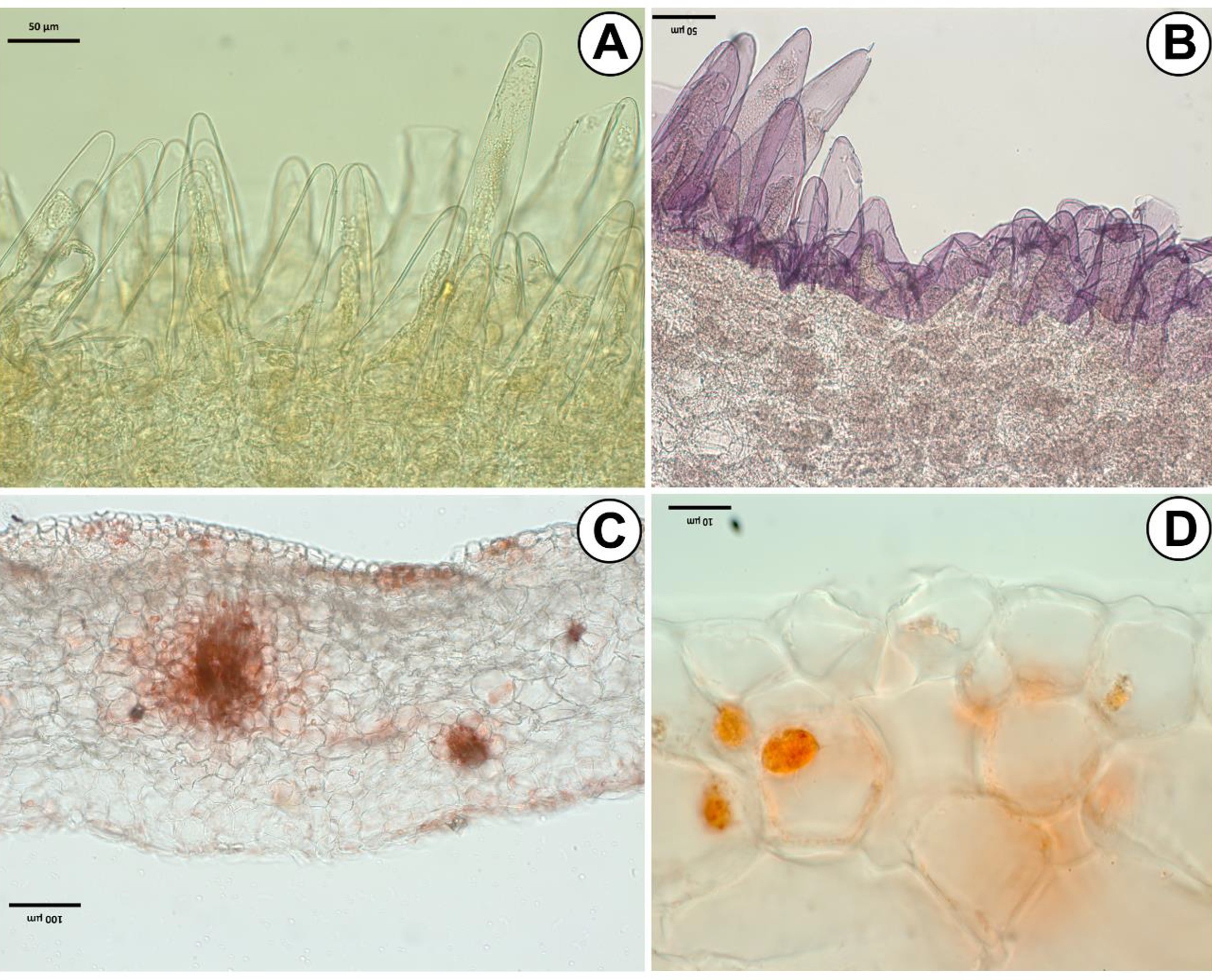

Supplement: plac039_suppl_Supplementary_Figure_S2 [file plac039_suppl_supplementary_figure_s2.jpeg]
