# Supplementary material for: Coryanthes macrantha (Orchidaceae: Stanhopeinae) and their floral and extrafloral secretory structures: an anatomical and phytochemical approach
Source: AoB Plants. 2022 Sep 2;14(5):plac039. doi: 10.1093/aobpla/plac039 (PMC9525647; doi:10.1093/aobpla/plac039)
Supplement: plac039_suppl_Supplementary_Table_S1 [file plac039_suppl_supplementary_table_s1.docx]

**Table S1**. Histochemical analyses of exsudates of the secreting structures of *Coryanthes macrantha*.

| **Test** | **Target Compounds** | **Reference** | **Result** | | |
| --- | --- | --- | --- | --- | --- |
|  |  |  | **Bract** | **Sepal** | **Labellum** |
| **Observed under visible light** | | | | | |
| Fehling's reagent | Polysaccharides | Sass 1951 | + | + | * |
| Lugol reagent | Starch | Johansen 1940 | + | + | + |
| Sudan III | Lipids | Johansen 1940 | - | - | + |
| Cooper acetate/rubeanic acid | Fatty acid | Ganter & Jolles, 1969, 1970 | * | * | + |
| Nile Blue sulfate | Acid and neutral lipids | Caim 1947 | * | * | + |
| NADI reagent | Terpenes | David & Carde 1964 | * | * | + |
| Xilidine Ponceau | Protein | Vidal 1970 | + | + | + |
| Ferric chloride 10% | Phenolic compounds | Johansen 1940 | - | - | - |
| Alcian Blue | Acidic mucilage and pectines | Perase 1985 | - | - | - |
| Ruthenium Red | Acidic mucilage and pectines | Gregory & Bass 1989 | - | - | - |
| **Observed unde the UV light** | | | | | |
| Neutral Red | Lipids | Kirk-Junior, 1970 | * | * | + |

(+) positive reaction; (-) negative reaction; (*) not apllied.
